# Neutrophil-to-lymphocyte ratio is an independent predictor for survival outcomes in cervical cancer: a systematic review and meta-analysis

**DOI:** 10.1038/s41598-020-79071-x

**Published:** 2020-12-14

**Authors:** Peijun Zou, E. Yang, Zhengyu Li

**Affiliations:** Department of Obstetrics and Gynecology, Key Laboratory of Birth Defects and Related Diseases of Women and Children, Ministry of Education, West China Second University Hospital, Sichuan University, No. 20 Section 3, Renmin South Road, Chengdu, 610041 Sichuan People’s Republic of China

**Keywords:** Prognostic markers, Cervical cancer, Cancer microenvironment

## Abstract

This updated meta-analysis sought to explore whether pretreatment neutrophil-to-lymphocyte ratio (NLR) could serve as an independent predictor for survival outcomes in patients with cervical cancer. We searched PubMed, Embase, Web of science and Scopus for studies on the association of pretreatment serum NLR with overall survival (OS) and progression-free survival (PFS) among patients with cervical cancer. Included studies with a hazard ratio (HR) and 95% confidence interval (CI) or a p-value were weighted by generic inverse-variance and pooled in a random effects meta-analysis. Subgroup analyses were conducted according to regions, NLR cut-off values and treatments. Publication bias was analyzed by Egger’s and Begg’s tests. A total of 14 studies comprising 6041 patients were included. The median cut-off value for NLR was 2.46 (range from 1.60 to 3.80). The higher NLR was associated to worse OS (HR 1.86, 95% CI 1.44–2.40) and PFS (HR 1.67, 95% CI 1.25–2.23), compared with lower NLR. This association still exited when analyzed according to regions, NLR cut-off values. Moreover, Significant association between NLR and OS was observed in studies which included patients with early stage disease and receiving radical surgeries. High NLR is independently associated with decreased OS and PFS in patients with cervical cancer. Pretreatment NLR is of independent value to predict the survival outcomes in patients with cervical cancer, regardless of regions and primary treatments.

## Introduction

Cervical cancer (CC) is the fourth most common female malignancy worldwide and remains a major global health challenge^1^. Actually, the disease is largely preventable by organized screening and human papilloma virus (HPV) vaccination programs. However, approximately 90% of patients with CC lived in low-income and middle-income countries that lack formal screening and HPV vacciniums. Despite of advances in the past 10 years, regionally different conditions in CC outcomes present an urgent need for more particular evidence-based management guidelines. More than half a million women are diagnosed with cervical cancer every year and over 300,000 women died from this disease all over the world, of which recurrence or progression takes the most blame^2^.

There has been some prognostic indicators used in cervical cancer, such as tumor size, lymph node status, histologic type and grade, lymphovascular space invasion (LVSI) and lymph node metastasis^3,4^. But most of them are acquired after surgery treatment and cannot be a feasible indicator for initial treatment. Therefore, it is urgent to identify accessible and feasible pretreatment indicators for prognostic surveillance, recognizing high-risk factors for recurrence, postoperative therapy and follow-up.

Some literatures demonstrated that approximately a quarter of advanced cancer patients with systemic inflammation have worse reaction to chemotherapy and also poorer survival outcomes, compared to those cancer patients without inflammation^5–7^. The challenge that we are confronted with is how to recognize the cancer-related systemic inflammation. Several studies identified some potential parameters in blood, including the neutrophil to lymphocyte ratio (NLR), derived NLR (dNLR), lymphocyte-monocyte ratio (LMR), platelet-lymphocyte ratio (PLR), elevated CRP and low albumin levels^6–9^. These biomarkers show a significant correlation and therefore may be implicating the further inflammatory processes. NLR^5^, defined as the ratio of neutrophil count to lymphocyte count in peripheral blood, is the most frequently reported in previous studies.

Presence of high NLR has been found to be correlated with worse outcomes in many malignancies, such as colorectal cancer, head and neck squamous cell carcinoma, soft tissue sarcoma, biliary tract cancers, ovarian cancer, gastrointestinal cancer and breast cancer^10–17^. A systemic dynamic balance was assumed between the systematic inflammation induced by tumor and the antitumor immune response derived from body, consisting of the neutrophil-dependent pro-tumor inflammation and the lymphocyte-associated anti-tumor immune response. This often manifests as neutrophilia, thrombocytosis and relative lymphocytopenia in the peripheral blood^18,19^.

Though, the elevated pretreatment NLR was supposed to be correlated to some gynecologic malignancies such as ovarian cancer, endometrial cancer and cervical cancer in previous literature^20,21^. However, the independent prognosis-predicting role of the pretreatment NLR in patients with cervical cancer is controversial, and the previous meta-analysis included a considerable number of studies without a multivariable analysis^22–24^, leading to unsure validity of the independent prognostic value of NLR. Therefore, we conducted this meta-analysis to elucidate the independent prognostic value of pretreatment NLR in CC.

## Materials and methods

### Search strategy

**Figure 1:**
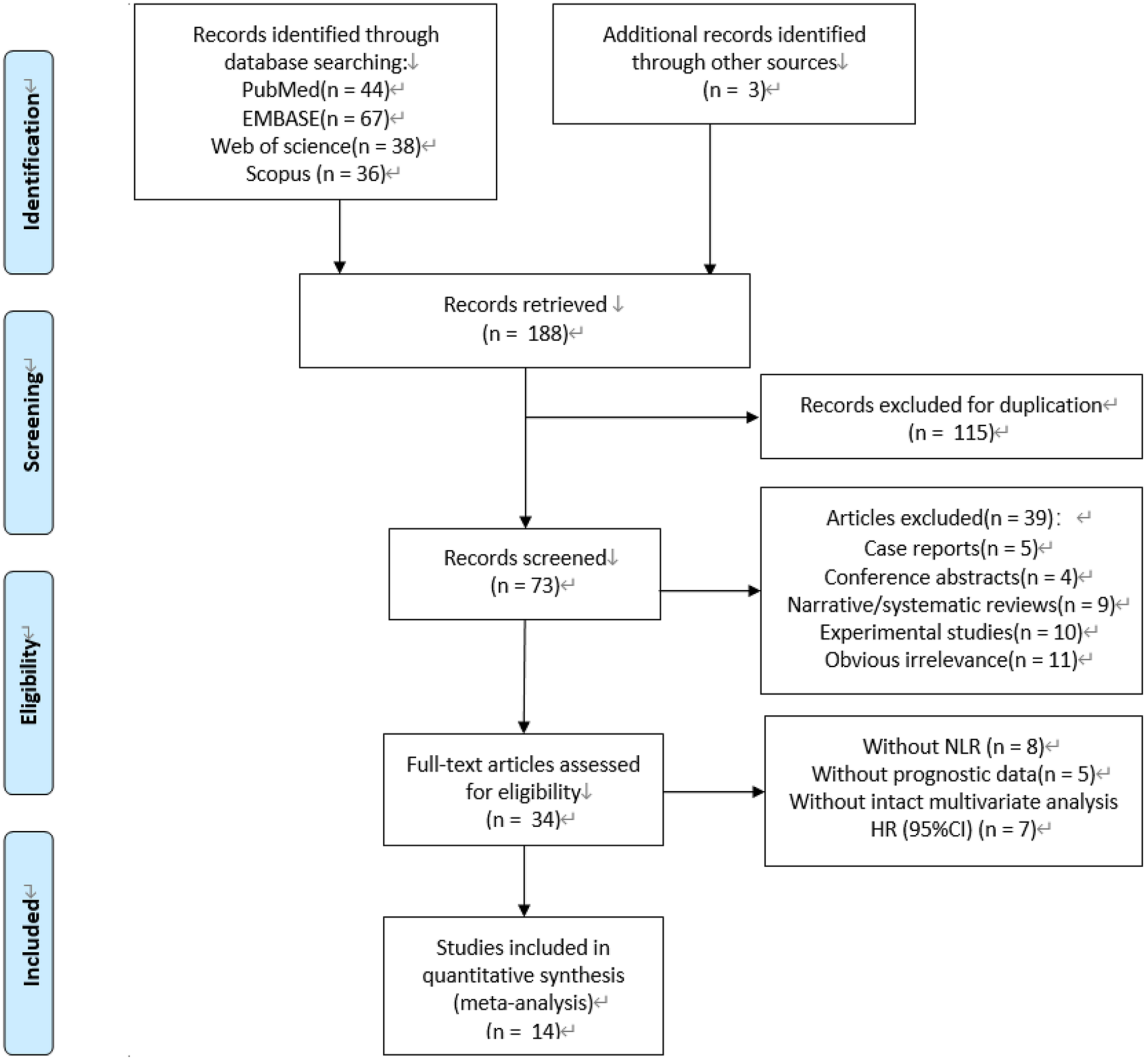
Search strategy and the flowchart of included studies.

This meta-analysis was reported according to the Preferred Reporting Items for Systematic Reviews and Meta-Analyses (PRISMA) guidelines^25,26^. A comprehensive electronic literature search was conducted through PubMed, Embase, Web of Science and Scopus databases, updated to January 15, 2019, based on the addition of following terms: “uterine cervical neoplasms” and synonymous cervical-cancer-specific terms, “NLR” or “neutrophil to lymphocyte ratio” or “neutrophil-to-lymphocyte ratio” or “neutrophil lymphocyte ratio”^27^. The references of retrieved articles were also screened manually for intact relevant studies. The full search strategy is described in Fig. 1.

### Study selection

All potential relevant articles were independently evaluate by two reviewers. All studies identified by the search were reviewed, and all candidate publications were retrieved in full text. Dissents were resolved by consensus. The inclusion criteria were as follows: (1) studies of adult women with cervical cancer confirmed by histopathology; (2) reported the association between NLR and the survival outcome of overall survival (OS) or progression-free survival (PFS); (3) the NLR from peripheral blood sampled prior to all treatment (surgery and/or systemic therapy and/or radiotherapy); (4) reported original data of a hazard ratio (HR) in multivariate analysis for OS, and/or PFS, and corresponding 95% confidence interval (CI) and/or p-value; (5) available as full-text publication; (6) clinical trials, cohort or case–control studies; (7) reported on English language publication. Studies were regarded as eligible when they satisfied all of the above criteria. Articles were excluded if they belonged to any of the following circumstances: (1) case reports, conference proceedings and letters to editors; (2) insufficient or ambiguous data to evaluate the HRs and 95% CIs; (3) overlapping or duplicate data; (4) Studies provided univariate data alone were excluded. When multiple publications or data analyses were likely from the same data source and without clarification on potentially duplicate data, the study reporting the largest number of patients was retained and other relevant studies were excluded. Corresponding authors were contacted on occasions to clarify missing or ambiguous data, but no additional data was received. Eight relevant articles identified in the previous systematic review were also included^22^.

### Data extraction

The detailed data were extracted from included studies in predesigned forms: (1) name of first author, (2) year of publication, (3) country of data source, further divided into Asian group and non-Asian group, (4) time of follow-up, (5) sample size of included analysis, (6) cut-off value used to define high NLR, (7) histology of patients included in analysis, (8) the FIGO stage of disease, (9) median or mean age, (10) median follow-up duration, (11) treatment strategies, (12) survival outcomes including OS and PFS and HRs from multivariate analysis with associated 95% CIs for OS and/or PFS. If all the patients in the individual study were treated with operative therapy with or without additional nonsurgical adjuvant therapy (AT) in the follow up, including chemotherapy (CT) and/or radiation therapy (RT), the study was classified into surgical ± AT subgroup. By contrary, if all the patients in the individual study received only non-surgical intervention such as RT and cisplatin-based concurrent chemoradiotherapy (CCRT), the study was classified into nonsurgical subgroup. Studies with obscured description for treatments were labelled as the Mixed therapy group.

### Assessment of methodological quality

The quality of included studies was evaluated by Newcastle–Ottawa Scale (NOS)^28^ according to the following categories: selection, comparability, and outcome of interest. The total score of NOS ranged from 0 to 9, and we considered studies as high quality if they met at least six scores (Supplementary Table 1).

### Statistical analyses

Extracted data were pooled using STATA version 14.0 analysis software (STATA Corporation, College Station, TX, USA). All statistical tests were two sided. A meta-analysis was conducted for all eligible studies and the estimated HR from multivariable analysis and 95% CI were used to assess the strength of association of NLR with survival endpoints. The primary outcome was overall survival (OS), meanwhile progression-free survival (PFS) and recurrence-free survival (RFS) were regarded as secondary outcomes which always shared equivalent definitions in included studies. Estimates for HRs were pooled and weighted by generic inverse variance. Heterogeneity assumption was examined by the Cochran’s Q static and Higgins I^2^ metric. P < 0.10 or I^2^ > 50% was considered as a measurement of extreme heterogeneity. A random-effects model (Der Simonian and Laird method) was performed to calculate the pooled HR estimation of each study when extreme heterogeneity existed. Subgroup analyses were conducted according to countries of datasets, cut-off values and treatment strategies. Potential publication bias was evaluated by Begg’s and Egger’s tests. A two-tailed P value of less than 0.05 was defined as statistically significance.

## Results

### Literature search and included studies

Fourteen studies comprising 6041 patients with cervical cancer were included^20,29–41^. Characteristics of included studies are described in Table 1. All of the qualified studies were observational retrospective studies. Three studies included only patients with early-stage disease whose primary treatment were radical surgery with or without adjuvant therapy, and one included only those with advanced disease received concurrent chemotherapy and radiation (CCRT). Ten studies included patients with both early and advanced disease, and of these, five studies included patients treated with radiation alone or CCRT. These groups divided by treatment strategy were considered separately. Four eligible studies targeted at non-Asian crowds, and ten studies were collected from Asian crowds. All the NLR in included studies were from peripheral blood sampled prior to all treatment.

**Table 1:** Characteristics of included studies.

Study	Year	Country	Time frame	Sample size	Age (median, year)	Histology	FIGO Stage	Follow-up duration (median)	Treatment strategy	Cut-off value
Jonska-Gmyrek et al	2018	Poland	2003–2008	94	53	ADC	IA–IVB	66 mons	Mixed	1.6
Nuchpramool et al	2018	Thailand	2001–2016	460	47	Mixed	IA2–IB1	57 mons	Surgery ± AT	1.8
Ida et al	2018	Japan	2004–2015	79	52.4^#^	Mixed	NR	15 mons	Mixed	2.8
Holub et al	2018	Spain	2009–2016	151	51	Mixed	IA–IVB	44 mons	Mixed	3.8
He et al	2018	China	2007–2009	229	44	Mixed	I–IV	83 mons	Mixed	1.6
Koulis et al	2017	Canada	1998–2012	257	50	Mixed	IB–IVA	41 mons	CCRT or RT	5
Cho et al.*	2016	Korea	1986–2012	2456	56	Mixed	IA–IVA	65 mons	CCRT or RT	2.5
Wang et al.*	2016	China	2009–2010	60	53	SCC	II–III	58 mons	CCRT	2
Onal et al.*	2016	Turkey	2006–2014	235	57	Mixed	IB2–IVA	32 mons	CCRT	3.03
Haraga et al.*	2016	Japan	2007–2013	36	61.5^#^	Mixed	IB1–IVA	30 mons	RT	2.85
Chen et al.*	2016	China	2006–2009	407	44	NR	IB1–IIA	NR	Surgery ± AT	2.42
Mizunuma et al.*	2015	Japan	2005–2013	56	65.1	SCC	IB1–IV	14 mons^#^	CCRT or RT	2.5
Zhang et al.*	2014	China	2005–2008	460	44	Mixed	I–II	69 mons	Surgery ± AT	2.213
Lee et al.*	2012	Korea	1996–2007	1061	50	Mixed	IB1–IVA	53 mons	Mixed	1.9

*NR* not reported, * ± AT* with/without adjuvant therapy, *CCRT* cisplatin-based concurrent chemoradiotherapy, *RT* radiotherapy, *ADC* adenocarcinoma of cervix, *SCC* squamous carcinoma of cervix, *FIGO* International Federation of Gynecology and Obstetrics.

^#^Mean age of the sample.

^#^Mean follow up duration of the sample.

*Included in previous meta-analysis.

### Overall survival

**Figure 2:**
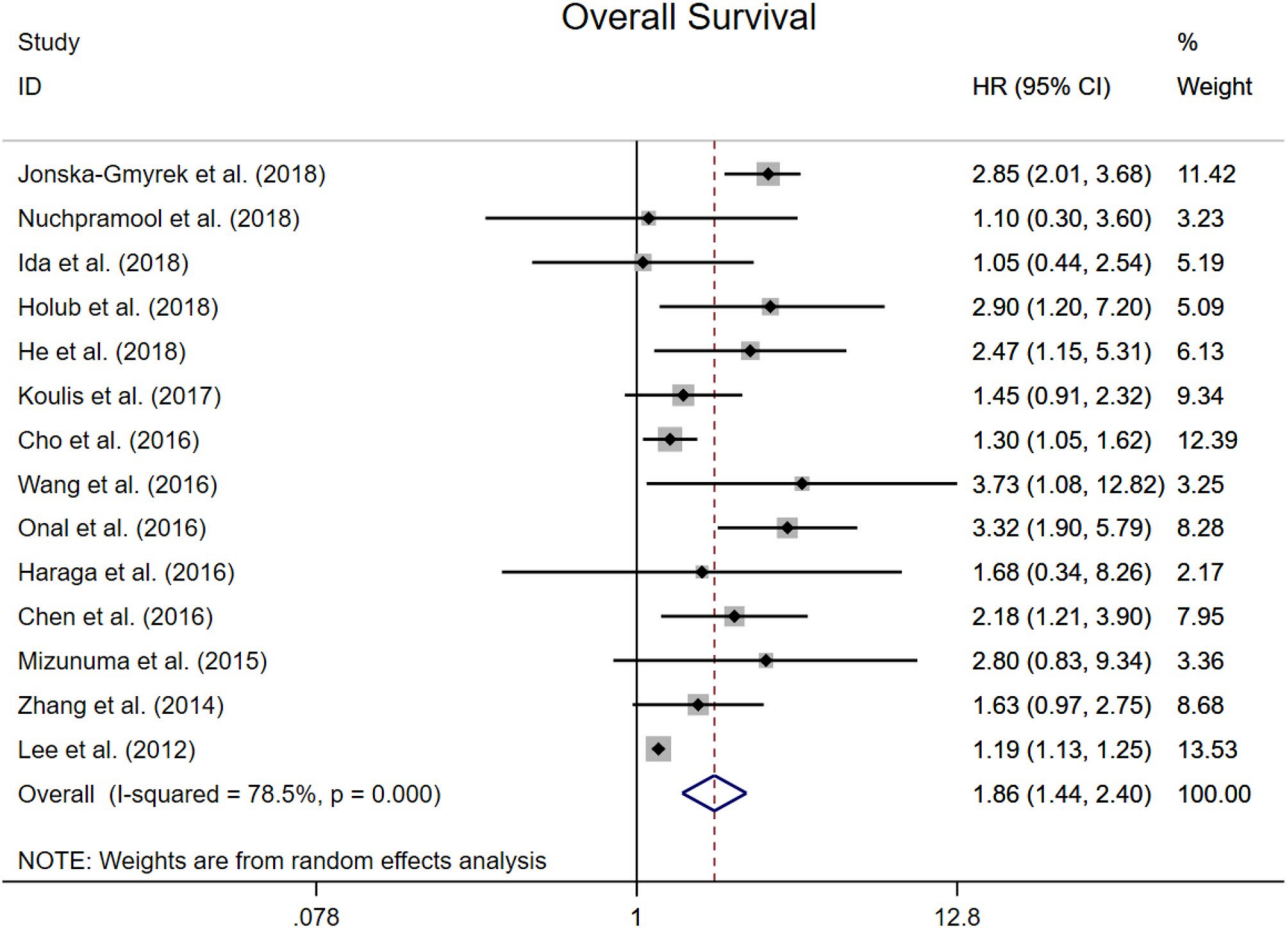
Forest plots showing hazard ratio for overall survival in all studies for neutrophil-to-lymphocyte ratio.

All studies reported adjusted HRs for OS. The median cut-off value for NLR was 2.46 (range from 1.60 to 3.80). Median (or mean) follow-up duration was reported in 13 studies, and ranged from 14 to 83 months (median inter-study 57 months). Overall, high NLR was associated with worse OS (HR 1.86, 95% CI 1.44–2.40; P < 0.001, Fig. 2). There was statistically significant heterogeneity (I^2^ = 78.5%), which was expected given the diversity of primary treatments and disease stage. Additionally, subgroup analyses showed that the region of dataset, primary treatment and NLR cut-off did not affect the association between high NLR and OS.

### Overall survival by the region of dataset

**Figure 3:**
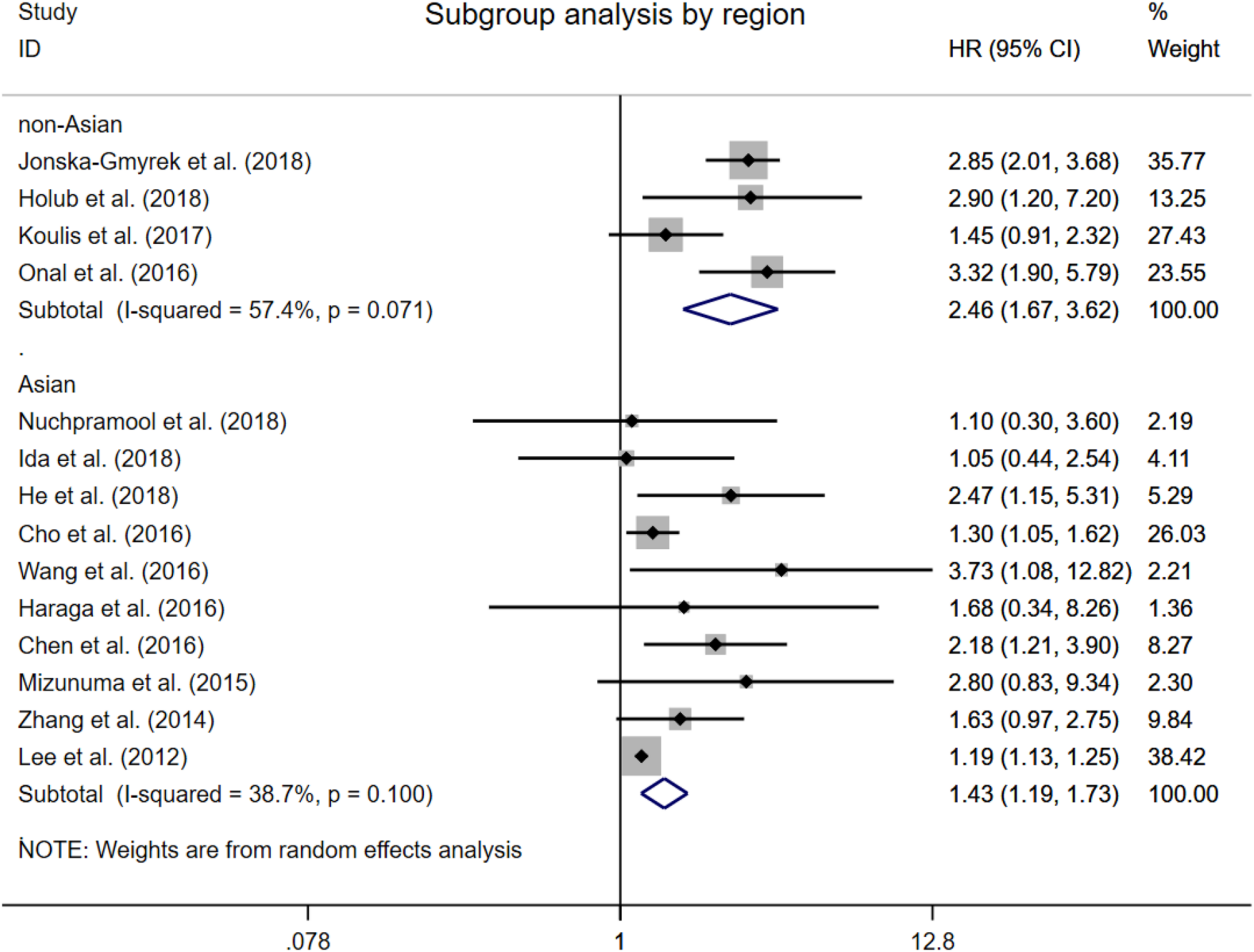
Forest plots showing hazard ratio for overall survival by regions in all studies for neutrophil-to-lymphocyte ratio.

In a subgroup analysis based on the region of dataset, the correlation of NLR greater than the cut-off with worse OS was maintained both in studies of Asian and non-Asian patients (Asian group: HR 1.43, 95% CI 1.19 to 1.73; non-Asian group: HR 2.46, 95% CI 1.67 to 3.62; Fig. 3). The presence of significant heterogeneity could be explained in a sensitivity analysis omitting studies from non-Asian group.

### Overall survival by primary treatment

**Figure 4:**
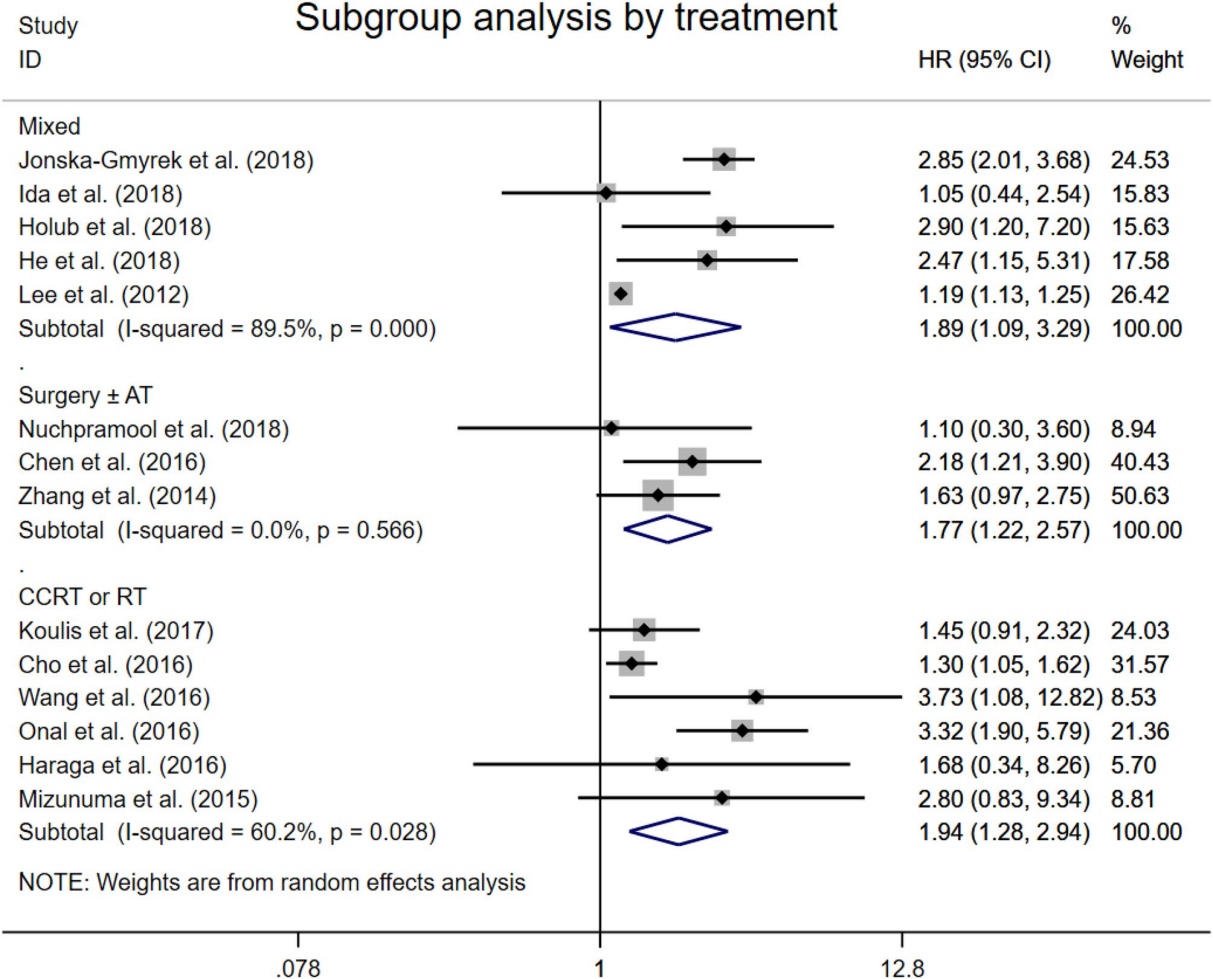
Forest plots showing hazard ratio for overall survival by treatments in all studies for neutrophil-to-lymphocyte ratio.

The combined analysis of three studies showed that patients with elevated NLR were expected to suffer unfavorable OS after surgery with or without any adjuvant therapy (HR 1.77, 95% CI 1.22–2.57, Fig. 4). In six studies defined CCRT or RT as the only primary treatment, significant association between high NLR and worse OS was observed (HR 1.94, 95% CI 1.28–2.94). The combined analysis of the rest five studies with mixed primary treatments drew the same conclusion (HR 1.89, 95% CI 1.09–3.29). In most eligible studies, the primary treatments of patients with cervical cancer were guided by the FIGO stage and treated according to European Society for Medical Oncology and European Society of Radiotherapy and Oncology (ESTRO) recommendations.

### Overall survival by cut-off value

**Figure 5:**
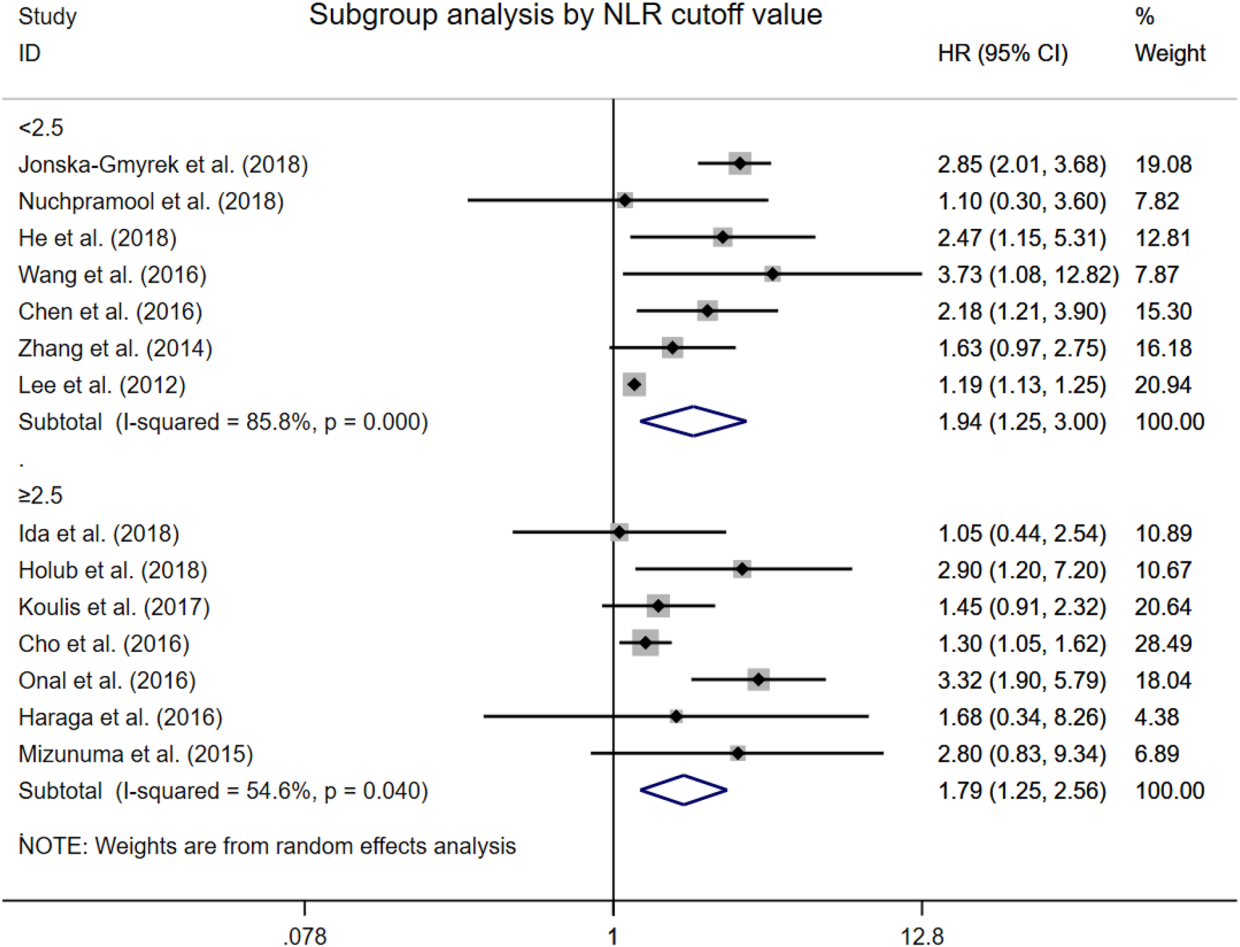
Forest plots showing hazard ratio for overall survival by cut-off values in all studies for neutrophil-to-lymphocyte ratio.

We defined the low NLR cut-off value group as the group including seven studies with NLR cut-off value lower than 2.5, which is according to the adjusted median NLR cut-off value of all eligible studies. To the contrary, the other seven studies were consisted of the high NLR cut-off value group. In subgroup analysis, both the low NLR cut-off value (HR 1.94, 95% CI 1.25–3.00, Fig. 5) and high NLR cut-off value (HR 1.79, 95% CI 1.25–2.56, Fig. 5) didn’t affect the prognostic effect of elevated NLR to poor OS. Similar to the other subgroup analysis for OS, exploratory analysis identified cervical cancer stage as a source of heterogeneity.

### Progression-free survival

**Figure 6:**
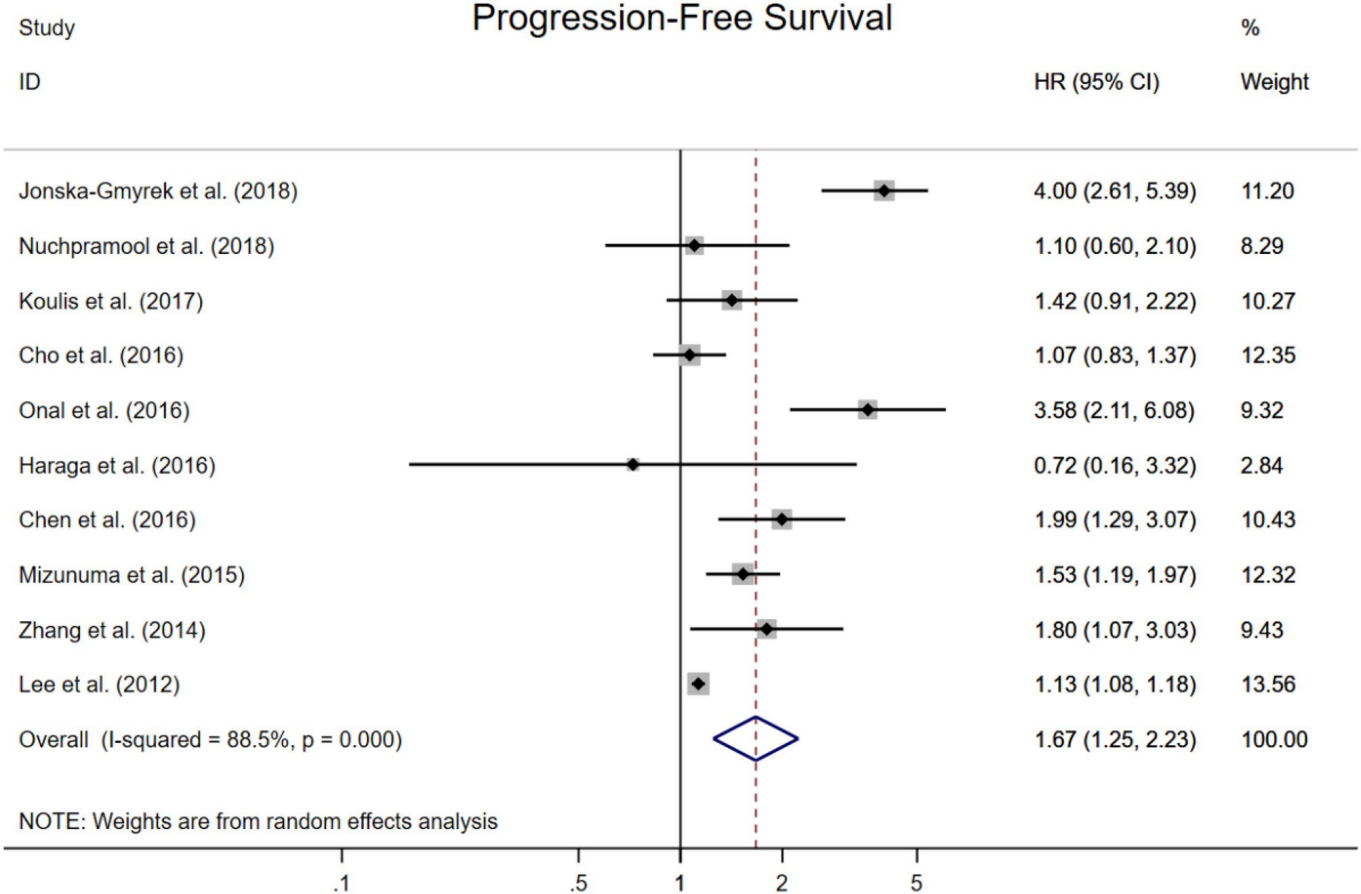
Forest plots showing hazard ratio for progression-free survival in all studies for neutrophil-to-lymphocyte ratio.

Ten studies provided HRs for PFS and the combined analysis showed a significant independent prognostic effect of elevated NLR to worse PFS (HR 1.67, 95% CI 1.25–2.23, Fig. 6). The median cut-off for high NLR was 2.46 (range 1.60–3.80). Median (or mean) follow-up was reported in 8 studies, and ranged from 14 to 83 months (median inter-study 57 months).

### Publication bias analysis

The funnel plots showed a low probability of publication bias (Supplementary Fig. 1). Consistently, the Begg’s tests demonstrated little evidence of publication bias for OS (P = 0.443) and for PFS (P = 0.721), respectively. However, the Egger’s tests showed a slight publication bias for OS (P = 0.006) and little bias for PFS (P = 0.054), given that fewer smaller studies reporting lower magnitude associations between NLR and OS.

## Discussion

Accumulating evidence have showed that elevated NLR might be related to cancer growth and metastasis^19,20,27,42^. It is associated with poorer response to chemotherapy and radiotherapy, worse survival outcomes^6^. Despite the mechanism why the increased NLR showed poorer outcomes is not completely clarified, neutrophils have been shown to suppress the activity of lymphocytes and T cell response, so that inhibiting the immune system^43^. In addition, through secreting high levels of proliferation factors such as chemokines, growth factors and circulating cytokines, neutrophils may also play a crucial role in orchestrating an inflammatory microenvironment which promotes growth, vascularization and metastasis of tumor. Besides, it was demonstrated by previous literature that the elevated NLR is associated with elevated tumor markers (CEA, CA19-9, and CA72-4), and lower infiltration of effective anti-tumor immune cells (lower CD3 and CD8 cytotoxic T cells) in the tumor microenvironment^44,45^.

Previous meta-analysis included both univariable and multivariable analyses, which allowed the results might be contaminated by other factors and weaken the independence of prognostic effect for NLR. To solve this problem, here we only included the studies with data of multivariable analysis^35–41^. Our results demonstrated an independent significant prognostic effect for elevated NLR on both poor OS and PFS, and this association was maintained across different regions, furthermore, NLR has the most prognostic value in Asian crowd with cervical cancer. The reason why we performed a sub-analysis on the regions is that previous studies have showed that the ethnic group and the regional economic and cultural level could influence the prognosis of cervical cancer. In addition, it has also been reported that some hematologic parameters, including NLR, differ in ethnic group. However, the detailed ethnic data of patients cannot be acquired in most included studies, we divided the group under the consideration of both ethnic group and the region. Our sub-analysis showed the lower heterogeneity in Asian group (I^2^ = 38.7%, Fig. 3), compared to non-Asian group (I^2^ = 57.4%, Fig. 3) and all studies group (I^2^ = 78.5%, Fig. 2), which may partly explain the source of the large heterogeneity in previous meta-analysis.

We showed that the elevated NLR also reveals a decreased OS in patients with CC in subgroup analysis by primary treatments and different NLR cut-off values. Furthermore, we investigated the association between NLR and other clinical factors in CC patients. We found that the pretreatment NLR was significantly associated with tumor grade, advanced FIGO stage, LVSI, LN metastasis and recurrence, which would influence the alternative of therapy strategies. These results are consistent with studies showing that neutrophilia and leukocytosis are associated to worse outcomes in patients with locally advanced and recurrent cervical cancer^29–31,46–48^.

We also noticed that different cut-off value of NLR in included studies might affect the final results and the validity, which was ignored in previous meta-analyses. In addition, the way to define the cut-off including ROC-AUC and mean/median, and the different lab condition where the hematologic parameter acquired, both alter the cut-off value. In our opinion, a valid NLR cut-off value plays an important role in studies, and that’s what we made up for the previous meta-analyses. The elevated NLR value were often defined greater than 5 in more than 60 studies comprising of over 37,000 patients with other cancers^6^. While in our study, the NLR more than 2.5 was identified high, given that the NLR cut-off value ranged from 1.60 to 3.80 (median 2.46) in the included studies. It is also noticed that the different cut-off value might lead to significant heterogeneity among studies. Thus, the definition of an elevated NLR or a consistent NLR cut-off value warrants further studies, which would investigate the distribution of NLR value and its relative influence factors among patients with cervical cancer.

The major reason why we didn’t conduct a sub-analysis on stage is that there were only three included studies focused on early-staged patients (Seen in Table 1: Nuchpramool et al.^29^ FIGO Stage: IA2-IB1; Chen et al.^38^ FIGO Stage: IB1–IIA; Zhang et al.^40^ FIGO Stage: I–II) and one focused on FIGO Stage II–III, while other studies reported mixed data of patients staged I–IV. As we all know, the initial treatment is mostly decided by the stage. In addition, we conducted a sub-analysis on treatment to explore its effect on NLR prognostic value, and we divided included studies into three group, of which the Surgery ± AT group was composed of the forementioned three studies with early-staged patients. We found a significant positive result and an extremely low heterogeneity (HR 1.77[1.22, 2.57], I^2^ = 0.0%, Fig. 4) in the Surgery ± AT/early-staged group, which imply that NLR may be better used for early-staged CC.

As a pretreatment routine in most clinical medical institution, the data of routine blood test from each individual is easily available. The feasibility, objectivity and quantifiability of pretreatment acquired hematologic parameters allow them become potential individual-oriented biomarkers, which are ready predictors for prognosis of patients with varied cancers. Among these parameters, NLR is the most widely studied and still remains the hot topic in the prognosis for many carcinomas. Till now, the application of NLR in clinic to predict cancer patients’ prognosis has been seen in other cancers such as UTUC^49^. But this vital parameter has not been included in the cervical cancer guidelines yet. That’s also one of the reasons why we conducted this meta-analysis. We intend to provided more evidence on this point and clarify whether it is suitable to apply this parameter in cervical cancer prognosis.

Although our study has demonstrated that elevated NLR is significantly associated with worse survival outcomes in cervical cancer, this study also has some limitations. First, meta-analyses based on published literature is potential to be biased towards positive results. Second, heterogeneity also remained in overall analyses. Third, prospective data are warranted to further validate the prognostic value of NLR in specific populations and clarify its role in disease pathogenesis. Last but not least, not only prognosis, but also the impact on inflammation and immune system warrants further investigation, since the host and tumor cells involved in the cancer-related systemic inflammation are both potential new prognostic predictors and drug targets in cancer chemotherapy^5,9,50,51^.

## Conclusions

High NLR is independently associated with decreased OS and PFS in patients with cervical cancer, and its prognostic value was showed among patients from different regions. Additionally, the different primary treatments and NLR cut-off value did not affect the association between high NLR and OS. However, the independent prognostic value of NLR for patients with cervical cancer still needs to be validated in prospective clinical trials. NLR is a readily accessible prognostic marker in the clinic pretreatment setting, and its applied cut-off value and impact on immune and targeted therapies warrants further investigation.

## Supplementary Information

Supplementary Information
