# Supplementary material for: Neutrophil-to-lymphocyte ratio is an independent predictor for survival outcomes in cervical cancer: a systematic review and meta-analysis
Source: Sci Rep. 2020 Dec 14;10:21917. doi: 10.1038/s41598-020-79071-x (PMC7736351; doi:10.1038/s41598-020-79071-x)

**Neutrophil-to-lymphocyte ratio is an independent predictor for survival outcomes in cervical cancer: a systematic review and meta-analysis**

Peijun Zou, E Yang, Zhengyu Li*

Department of Obstetrics and Gynecology, West China Second University Hospital, Sichuan University, No. 20 Section 3, Renmin South Road, Chengdu, Sichuan 610041, People’s Republic of China.

***Correspondence:**

Zhengyu Li, M.D. Department of Obstetrics and Gynecology, Key Laboratory of Birth Defects and Related Diseases of Women and Children, Ministry of Education, West China Second University Hospital, Sichuan University, No. 20 Section 3, Renmin South Road, Chengdu, Sichuan 610041, People’s Republic of China. (Telephone: +86-189-8215-1025; FAX: +86-28-8550-2391; Email: [zhengyuli@scu.edu.cn](mailto:zhengyuli@scu.edu.cn))

**Supplementary Table 1** NOS for included studies

| Study | Adjusted for | NOS |
| --- | --- | --- |
| Jonska-Gmyrek et al. | NA | 8 |
| Nuchpramool et al. | 1,18,19,22(PFS);1,3,22(OS) | 8 |
| Ida et al. | 1,2,16,17,23(OS) | 7 |
| Holub et al. | 1,7,8,9,13,14,15(OS) | 8 |
| He et al. | 10(in SCC subgroup),11,15,16,22(OS) | 7 |
| Koulis et al. | 3,15,18,22(PFS);3,13(OS) | 7 |
| Cho et al. | 3,5,6,13,14,15,16,18,22(PFS);3,5,6,13,14,15,18,22(OS) | 8 |
| Wang et al. | 15,22(OS) | 7 |
| Onal et al. | 1,14,15,13,22(PFS,OS) | 9 |
| Haraga et al. | 2,14,15,20,22(PFS,OS) | 8 |
| Chen et al. | 1,4,12,19,21,22(PFS);1,12,15,19,22(OS) | 8 |
| Mizunuma et al. | 3,14,15,16(PFS,OS) | 9 |
| Zhang et al. | 15,22(PFS,OS) | 8 |
| Lee et al. | 13,15,16,18(PFS,OS) | 8 |

Abbreviations: NOS Newcastle Ottawa Scale

**Supplementary Fig. 1**


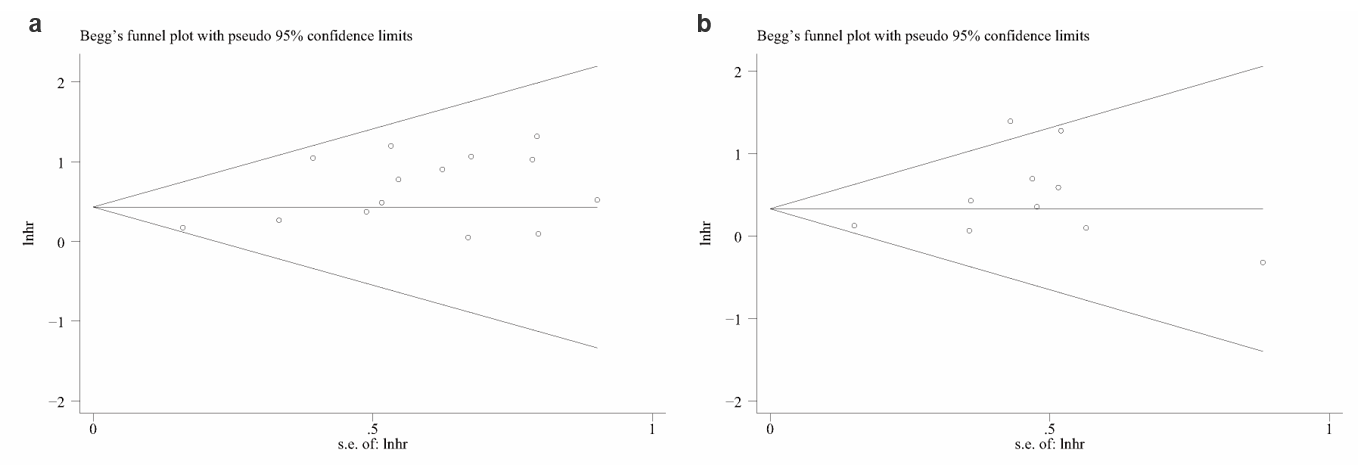

Supplement: Supplementary file 1 — Supplementary Information [file 41598_2020_79071_MOESM1_ESM.docx]
